# MANAGING DEATH IN THE FIELD: HOW EMERGENCY MEDICAL SERVICES TEAMS PROVIDE END-OF-LIFE CARE

**DOI:** 10.1093/geroni/igz038.1847

**Published:** 2019-11-08

**Authors:** Deborah P Waldrop, Jacqueline M McGinley, Brian M Clemency

**Affiliations:** 1 University at Buffalo School of Social Work, Buffalo, New York, United States; 2 Buffalo State College, Buffalo, New York, United States; 3 Erie County Medical Center, Buffalo, New York, United States

## Abstract

Emergency medical services (EMS) providers respond more frequently to calls for older adults with serious illness than for people in other age groups. Recent legislation that makes it possible to document healthcare decisions has facilitated an era of choice in end-of-life care. EMS teams make time-sensitive decisions about care, resuscitation and hospital transport that influence how and where a seriously ill older adult will die and how his/her family will experience the death. Yet, EMS providers’ perspectives on urgent decision-making and how they work with families are unknown. The purpose of this study was to explore the decision-making process that occurs how EMS teams respond when someone is dying from a serious illness (vs. an injury). In-depth in-person interviews were conducted with 50 EMS providers (24 emergency medical technicians [EMTs] and 26 Paramedics) from four ambulance services. Participants’ ages ranged from 21-57 (M=37.9) and 70% were male. Qualitative data was coded using Atlas.ti software. Three themes illuminated participants’ experiences with end-of-life calls: (1) How legally binding documents (e.g. Do Not Resuscitate [DNR] orders, Medical Orders for Life Sustaining Treatment [MOLST]) inform care; (2) Decision-making about foregoing or halting resuscitation (e.g. no hospitalization, death at home); and (3) Family care, support and education. The results suggest that EMS providers have critically important roles in upholding the wishes of seriously ill older adults and helping caregiving families through the end-of-life transition. Implications: Discussions about the meaning of legally binding documents (e.g. DNR, MOLST) and EMS calls are important in advance care planning.

